# Adapting a digital quality improvement system (Neotree) for newborn care in primary health centres and community hospitals: a mixed-methods implementation study in Malawi

**DOI:** 10.1080/16549716.2025.2560716

**Published:** 2025-10-23

**Authors:** Emma Wilson, Tim Hull-Bailey, Sophie Sutcliffe Goodman, Mtheto Sinjani, Elizabeth Chintolo, Rashid Deula, Kondwani Mwandira, Deliwe Nkhoma, Gloria Zailani, Aditi Rao, Msandeni Chiume, Michelle Heys

**Affiliations:** a Population, Policy and Practice, UCL Great Ormond Street Institute of Child Health, London, UK; b Neotree Charity, London, UK; c Global Health Informatics Institute, Lilongwe, Malawi; d Department of Paediatrics, Kamuzu Central Hospital, Lilongwe, Malawi; e Malawi Epidemiology and Intervention Research Unit (MEIRU), Lilongwe, Malawi; f Specialist Children & Young People’s Services, East London NHS Foundation Trust, London, UK

**Keywords:** Digital health, newborn care, adaptation, primary health care, implementation research

## Abstract

**Background:**

Digital innovations incorporating clinical digital support can improve the delivery of perinatal and postnatal care, yet few interventions exist in primary care settings.

**Objectives:**

We aimed to adapt a digital quality improvement system (Neotree) from tertiary facilities to lower-level facilities providing Level 1 newborn care.

**Methods:**

We conducted a mixed method stepwise study using the ADAPT framework. We conducted a needs and technology assessment of eight facilities in Lilongwe district, Malawi. We next adopted a user-centred approach to modify the clinical decision support and data capture functionalities to fit the new context. We completed ‘think aloud’ usability testing with six prospective users to refine the system, alongside qualitative research informed by behavioural science frameworks with 10 healthcare professionals to identify potential barriers and facilitators to implementation. Finally, we carried out a stakeholder assessment to identify a potential pathway to scale.

**Results:**

The adapted application was highly usable achieving a mean System Usability Scale (SUS) score of 92.5 among prospective users during the final round of testing. Our qualitative findings indicated Neotree was anticipated to be acceptable among healthcare professionals. We found high levels of motivation to implement Neotree, but key perceived implementation barriers included psychological and physical capability (such as skills and knowledge in neonatal care), as well as physical opportunity (e.g. human resources, equipment and adequate space for newborn care).

**Conclusion:**

Using a stepwise user-centred approach, we successfully adapted a digital quality improvement intervention (Neotree) ready for real-world piloting in community hospital and primary health centres in Malawi.

## Paper Context

- **Main findings:** Despite the dearth of digital data capture and clinical decision support systems for newborn care outside of tertiary settings, we found healthcare professionals in lower-level facilities were highly motivated to use an adapted digital quality improvement system to support the delivery of newborn care.
- **Added knowledge:** In our intervention adaptation process, we found that lower-level facilities providing basic essential newborn care in Malawi are falling short of global standards, with respect to training, routine data collection and basic equipment.
- **Global health impact for policy and action:** International guidance for the provision of newborn care across all levels of the health system need to be adopted within national contexts, thereby ensuring implementers have contextually relevant benchmarks to achieve quality improvements across the care continuum.

## Background

Preventable newborn mortality remains a persistent global health problem which disproportionately impacts women, families and communities in low resource settings. Worldwide, 2.3 million children younger than 28 days die every year, representing 47% of deaths in children under five [1]. While most women (~80%) deliver in healthcare facilities, this has not translated into expected reductions in neonatal deaths [2]. In 2022, 64 countries were not on track to meet the Sustainable Development Goal to reduce neonatal mortality to 12 deaths per 1,000 live births [1].

Women’s access to facility-based care is far from uniform. An estimated 40% of women in sub-Saharan African countries deliver in primary care facilities, where quality of care is significantly lower than secondary or tertiary level facilities [3–5]. This variation in quality is linked to disparities in clinical outcomes in both obstetric and newborn care [3]. A recent study based on Malawian data found that facilities classed as higher quality (mainly hospitals) were associated with 23 less neonatal deaths per 1,000 births than lower quality facilities (after adjusting for maternal characteristics and facility selection based on risk) [6]. There is increasing recognition that a holistic, system-wide approach is required to improve the quality of perinatal and postnatal care across the care continuum if mortality targets are to be met [3,4].

System-wide approaches to quality improvement require clear delineation of care processes at each tier of the health system (i.e. community, primary, secondary and tertiary) [7]. This can allow for appropriate benchmarking and oversight of institutional capacity across the system to deliver the correct level of care [8], alongside careful monitoring of communication and referral processes between facilities. However, the World Health Organisation (WHO) has only recently produced international guidance on recommended levels of neonatal care [9]; and in many low-income settings, categorisations of neonatal care have yet to be specified within national policies and frameworks [10]. At a minimum, any facility where women deliver, including primary facilities, should provide a basic package of evidence-based interventions (Level 1 care). This includes essential newborn care (i.e. drying, hygiene cord care, delayed cord clamping, skin-to-skin contact, Vitamin K), thermal protection, early initiation and support for breastfeeding, resuscitation (where needed) and Prevention of Mother to Child Transmission of HIV [7,9]. Secondary and tertiary level facilities, such as district and central hospitals, should enable specialist in-patient care for sick, preterm and low-weight babies (Level 2 care); and depending on context and resources, tertiary facilities may also be equipped to provide round-the-clock intensive neonatal care such as mechanical ventilation (Level 3 care) [9] (see Supplementary File 1 for WHO levels of newborn care).

In many low resource contexts, the provision of quality newborn care, even the most basic essential care, is found lacking [5,6]. Key barriers include staff shortages, limited training and education, lack of basic supplies and infrastructure, weak clinical leadership, and low adherence to evidence-based guidelines [11]. Quality routine data are sparse, particularly outside of tertiary settings. Such data are vital for surveillance, quality benchmarking and monitoring, as well as enabling epidemiological research to inform the development of contextually relevant clinical guidelines, training and education [12].

Digital innovations are increasingly recognised for their potential to accelerate health systems strengthening, by enhancing both the quality and coverage of evidence-based interventions [13,14]. These include electronic capture of routine data [12], digital clinical decision support [15] and low-dose, high-frequency, training and education [13]. Yet progress on developing and embedding these tools within low-resource clinical settings has been piecemeal and evidence of successful implementation at scale is lacking [16]. For example in many low-resource settings, national electronic health record (EHR) systems, with potential for integrated clinical decision support functionalities, are still in the pilot stage [17].

### Neotree – a quality improvement digital intervention for neonatal care

**Figure 1.**
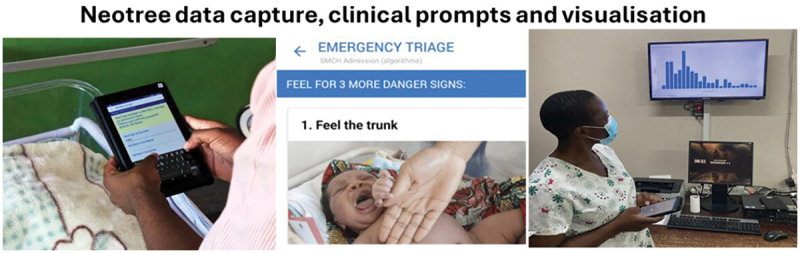
Neotree data capture, clinical prompts and visualisation.

Since 2014 we have been co-developing our intervention – Neotree – with Healthcare Professionals (HCPs) of all cadres within hospital facilities providing either level 2 or level 3 newborn care. Neotree combines electronic data capture, clinical decision support, training, and education within a single platform for newborn care, facilitating cycles of continuous learning and quality improvement (Figure 1) [18,19]. On admission, HCPs enter data onto an android-based application, which triggers educational messages and prompts (e.g. check the baby’s oxygen levels). These data feed into evidenced-based algorithms within the application which guide the user through diagnoses and management guidelines. An individual record of care is printed and added to patient notes, although a daily digital record of care is under development. Pseudonymised data are exported to a local database, visualised on data dashboards, and made available for audit and quality improvement initiatives. Data can be linked directly to national data systems (e.g. DHISv2 and the Zimbabwean Impilo Electronic Healthcare Records system).

The platform has supported the care of over 35,000 babies, by more than 1,200 healthcare professionals, in three hospitals across Malawi and Zimbabwe, demonstrating high feasibility and acceptability [20,21]. Our emerging evidence base is promising. The technology has supported quality improvement, leading to a reduction in prescription of unnecessary antibiotics and reduced rates of hypothermia on admission [22,23]. Cost analyses have demonstrated that Neotree is a time and cost-efficient tool, comparable with similar mHealth decision-support tools in low resource settings [24]; and one quasi-experimental study from a nurse-led site in Zimbabwe has indicated a reduction in mortality among low-weight babies, and an incremental cost-effectiveness ratio (ICER) of 6.35 USD per healthy life year gained, assuming implementation at scale [25].

### Rationale for adapting Neotree to lower-level facilities

Given the stagnation in progress towards global maternal and neonatal mortality targets, there are calls for a radical redesign of health systems to ensure that all women can deliver in advanced facilities capable of emergency obstetric care (i.e. C-sections) [5,26,27]. This may have the greatest impact on neonatal mortality [28], but in contexts such as Malawi, this strategy would require large-scale investments in infrastructure, personnel, and transport. Evidence from Malawi also suggests that women prefer to deliver in facilities that are free and close to home regardless of standards of care [29] and therefore a move to advanced facilities may require a shift in cultural norms and practices. Until large-scale investments are feasible, many women will continue to deliver in lower-level facilities, where interventions to improve quality of care are urgently needed [4]. Moreover a focus on primary care has equity implications, given that women delivering in these facilities are disproportionately from rural areas, and often poorer than women who deliver in secondary and tertiary centres [5].

In 2022, senior clinicians and stakeholders at the Ministries of Health in both Malawi and Zimbabwe asked the Neotree team to investigate whether the platform could be adapted to support health care professionals to capture data and deliver newborn care in primary care settings. This was in response to Neotree data from tertiary facilities which indicated that out-born babies (i.e. those delivered in primary health centres and transferred into tertiary centres) had worse clinical outcomes than in-born babies [23]. To our knowledge there are no similar digital platforms in operation in low resource settings [19].

Our working hypothesis is that Neotree is a suitable candidate for adaptation. The intervention was co-developed with, and for, all cadres of HCPs including those with limited neonatal training. The content of the app is easily configurable via a web-based editor, thus ensuring that the data capture functionality and associated algorithms (diagnoses and management) can be tailored to suit the specific clinical setting [18]. The system runs on low-cost hardware – in tertiary settings this includes one android tablet per 50 monthly admissions and one printer per unit – alongside open-source software. Neotree can cope with interruptions in power and internet connectivity. For example, data can be entered into the app offline and synced with a database (i.e. on a cloud-based server) once internet connection is available, while basic hardware such as tablets and printers can run on an intermittent power supply. Full specifications of the system have been described elsewhere [18].

## Methods

### Study aim

We aimed to adapt a digital quality improvement system (Neotree) from tertiary facilities to lower-level facilities providing Level 1 newborn care in Malawi.

### Study design

**Figure 2.**
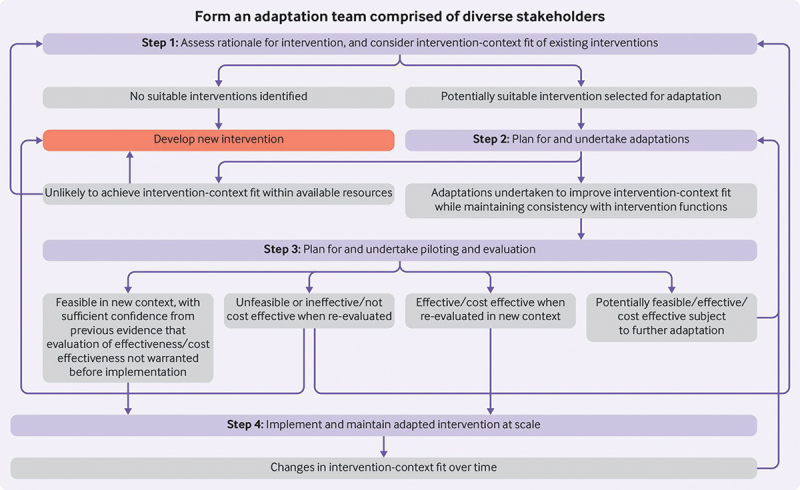
Adapt framework [30].

We conducted a mixed methods intervention adaptation study following the ADAPT framework for modifying evidenced-based interventions for new settings and populations [30]. This framework describes four key steps in the adaptation process (Figure 2). We describe the rationale for adaptation above (ADAPT step 1), and in the following sections we report the process of adapting Neotree to ensure a good fit with the new clinical context, while maintaining consistency with the original intervention functions (ADAPT step 2).

Key questions for this stage in the adaptation included:

1. What changes are required to the data capture and clinical decision support functionalities within the Neotree app to support the delivery of care in lower-level facilities?
2. Do lower-level facilities have sufficient resources and infrastructure to support Neotree intervention?
3. What implementation strategies are required to pilot Neotree in lower-level facilities?
4. Is Neotree likely to be acceptable, feasible and sustainable in lower-level facilities?

Next steps will be to deliver a version of Neotree informed by evidence and experience that is ready for pilot evaluation and rollout in lower-level facilities across Malawi (ADAPT steps 3 and 4).

#### Setting

Malawi is a resource-poor country in sub-Saharan Africa. Healthcare services are provided through the government (free at the point of care), the Christian Health Association of Malawi (with a small user fee), and other private or not-for-profit facilities, contributing 68%, 29%, and 3% of care, respectively [31]. The government’s healthcare provision includes four central hospitals delivering the highest level of care, 24 district and 25 community/rural hospitals, and approximately 430 primary health care centres [32]. Between 1990 and 2015 the country achieved a two-thirds reduction in childhood mortality [33]. However, neonatal mortality remains high at 18.7 per 1,000 live births in 2022 [34].

The focus of this adaptation work is Lilongwe district in the central region of Malawi, where our current intervention site, Kamuzu Central Hospital, acts as a referral hub providing the highest level of neonatal care [Level 2 care] within a linked network of primary health centres, community hospital and district facilities.

**Figure 3.**
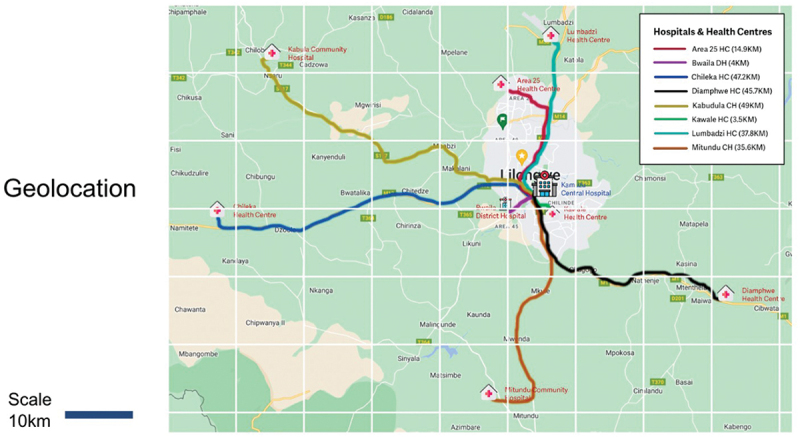
Location of health facilities in relation to Kamuzu Central hospital.

We selected a purposive sample of facilities, which were accessible by car from the capital of Lilongwe and within 500 metres of the main road. We selected a range of facilities providing level 1 newborn care, to gauge the feasibility of, and requirements for, adapting the platform across the network (Figure 3). Selected facilities varied by size, location (e.g. rural/urban) and capacity. They included two community hospitals, three urban/semi-urban primary health centres and two rural primary health centres (Table 1). We also included Bwaila district hospital, which provides level 2 newborn care, as it is a key referral centre receiving cases from primary and community hospital facilities. The Bwaila clinical team therefore had an appreciation of the bottlenecks and quality gaps within the referral network. Facilities were categorised as providing level 1 or level 2 newborn care in accordance with available guidance and expert opinion from within the adaptation team [35].

**Table 1.** Facilities selected to inform Neotree adaptation.

Level 2 newborn care	Level 1 newborn care		
District Hospital	Community Hospital	Urban and/or Semi-urban Health centres	Rural Health Centres
Bwaila District Hospital	Mitundu Community Hospital	Lumbadzi Health centre	Diamphwe Health centre
	Kabudula Community Hospital	Kawale Health centre	Chileka Health centre
		Area 25 Health centre	

#### Adaptation team

The adaptation team included a broad range of stakeholders from the Neotree charity, University College London, the Global Health Informatics Institute (Neotree’s main implementing partner in Malawi), Kamuzu Central Hospital (KCH), Malawi, and Baobab Web Services, South Africa. They included clinical and nursing staff, programme and implementation managers, software developers, implementation scientists, senior paediatricians and senior investigators. The team also received technical guidance from the UK Foreign, Commonwealth & Development Office (FCDO), UNICEF and the Ministry of Health in Malawi.

#### Ethics

Ethical Approvals were granted by Malawi College of Medicine Research and Ethics Committee (Ref: P.01/20/2909) and from University College London (Ref: 17123/001).

## Results

We conducted four key activities in a stepwise fashion with the output from each activity informing each subsequent step. These were:

(1) Health and technology needs assessment
(2) Adaptation of the content of the Neotree platform.
    (2.1) Agreement of a minimum data set of clinical and demographic variables for lower-level facilities.
    (2.2) Adaptation of the clinical content within the Neotree application to fit the new clinical context.
    (2.3) Usability testing to test and refine the application with prospective end-users.
    (2.4) Workshop to explore modifications to the data dashboard with prospective end-users.
(3) Qualitative research to explore anticipated acceptability and potential barriers and facilitators to implementing the adapted platform.
(4) Stakeholder consultation to explore sustainability and implementation strategies at scale.

In the following sections we describe the methods and findings for each of these activities.

## Health and technology needs assessment

### Methods

In October 2022, we conducted a health and technology needs assessment at eight selected facilities to understand the clinical management of neonates in these settings, the available infrastructure and resources, and the use of health information (Supplementary File 2). The site visits were conducted by a multidisciplinary team (*n* = 7) of clinicians and nurses from Kamuzu Central Hospital, and project managers from Global Health Informatics Institute. Face-to-face interviews were conducted with clinical officers in charge, nurses, administrators and data management personnel where available. The interviews lasted 1–2 hours, followed by a tour of the facilities.

### Findings

#### Staffing and training

In all facilities, postnatal care was nurse-led. At the district hospital oversight was provided by a paediatrician (registrar) while at the community hospitals and urban/semi urban PHCs, oversight was provided by a clinical or medical officer with responsibility for medical care across the whole facility. The two rural PHCs were run solely by nurses [Supplementary File 3].

Training in essential newborn care was lacking, and staff described a skills gap in basic clinical procedures such as newborn resuscitation. Staff at the district hospital had completed the national Care of the Infant Newborn (COIN) training but none had received refresher training. Staff at four facilities had not received any in-service training in essential newborn care. Many facilities reported staff shortages. The PHCs described the lack of nurse escorts to accompany sick and small neonates during transit to a referral centre, as a particular challenge.

#### Complexity of care

The district hospital, two community hospitals and one primary health centre reported capacity to conduct emergency obstetric care (C-sections). The district hospital is a key referral centre both for obstetric and newborn care serving two community hospitals and 56 health facilities in Lilongwe district. It conducts an estimated 1555 deliveries per month and has capacity for 47 small vulnerable neonates in the nursery [Supplementary File 4]. It provides most newborn interventions, but very complex cases including those requiring surgical care are referred to KCH.

**Figure 4.**
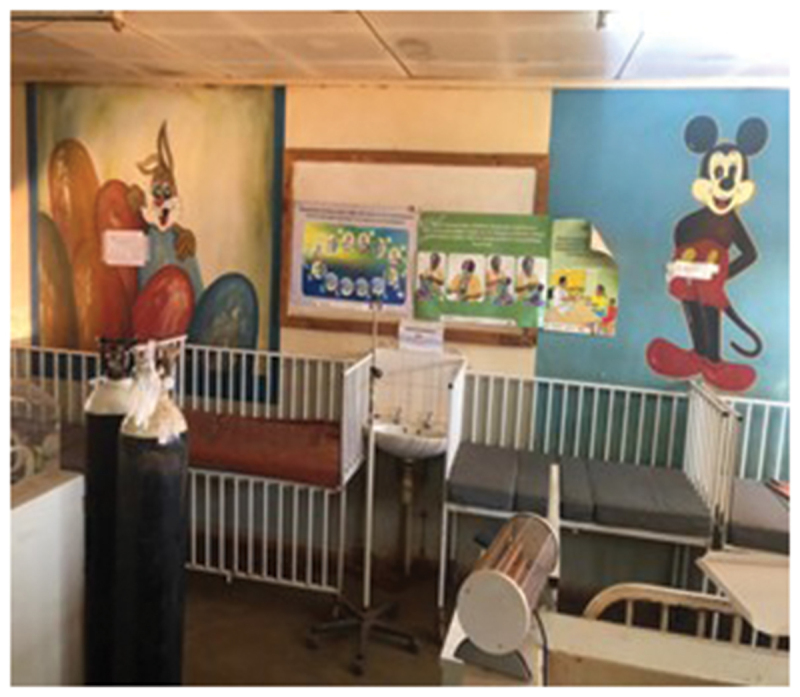
Nursery in a community hospital.

The two community hospitals reported the provision of limited in-patient newborn care, such as hospital-based Kangaroo Mother Care (KMC), and the treatment of mild infections for babies over 1.5 kg (Figure 4). As expected, most primary health centres had no in-patient capacity. If presented with a sick or small neonate, HCPs try to stabilise and transfer the patient to a larger centre.

#### Equipment for newborn care

**Figure 5.**
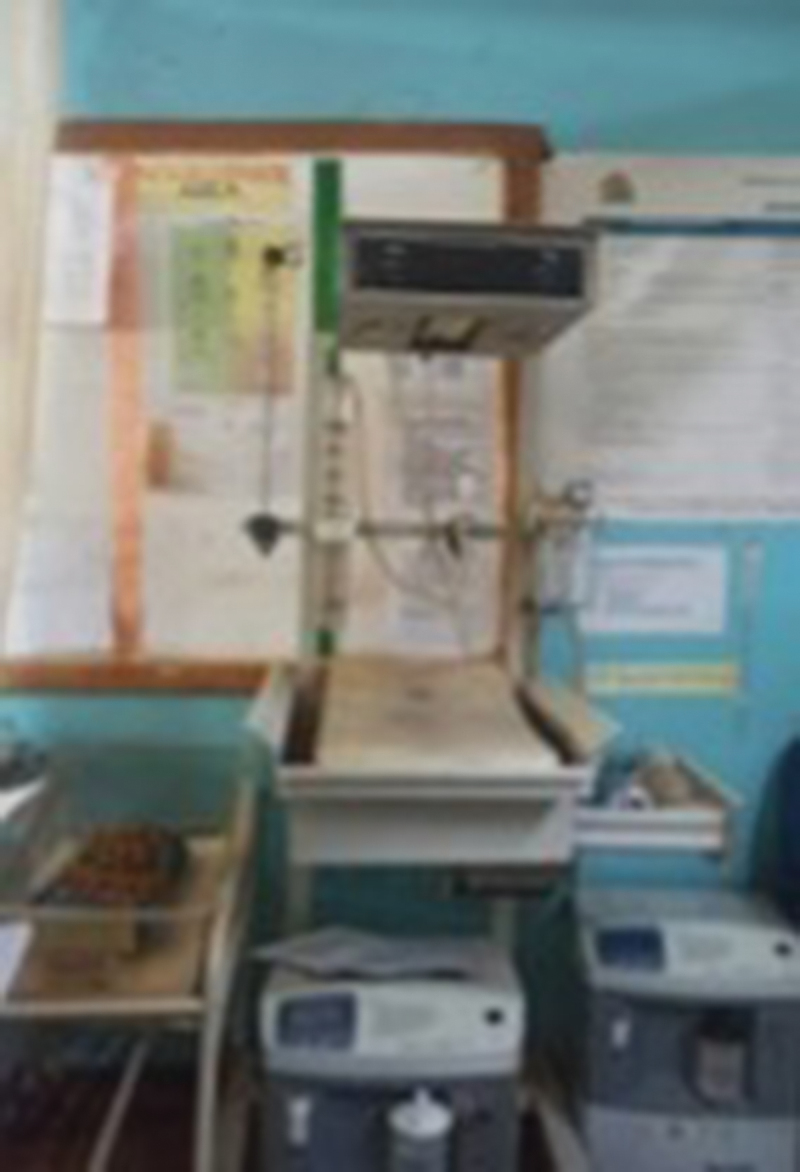
Newborn care corner in a community hospital.

**Figure 6.**
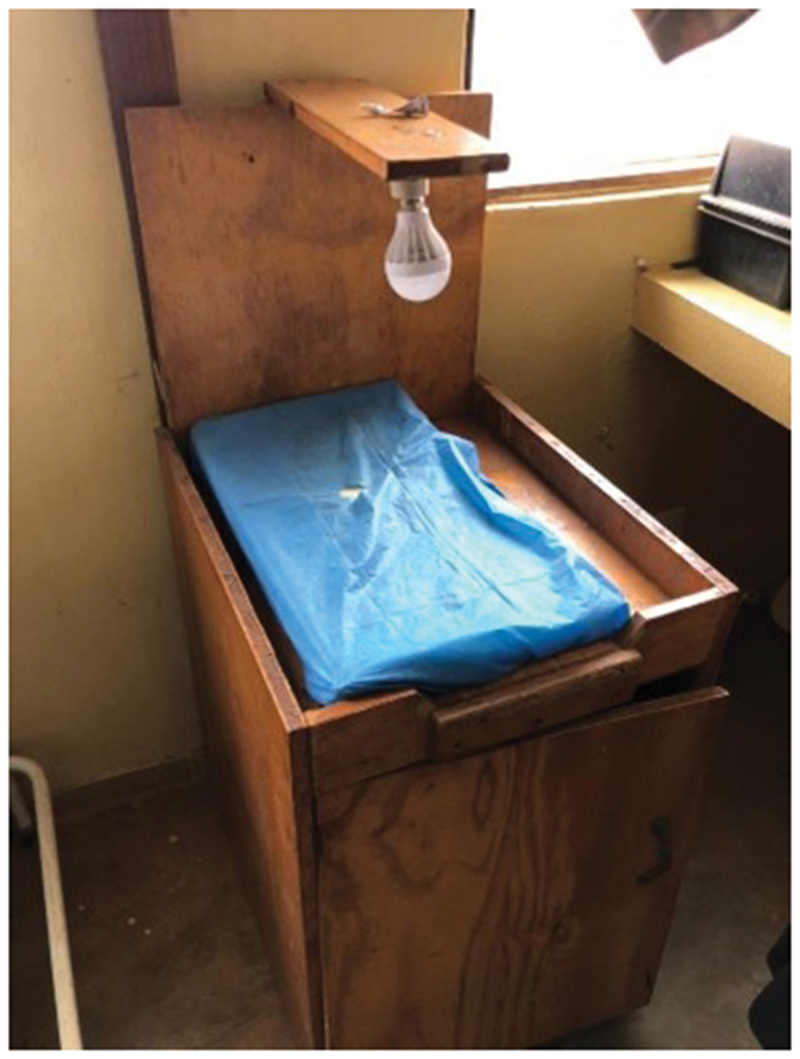
Newborn care corner in a rural primary health centre.

Table 2 shows the available equipment at each facility, categorised by newborn intervention. All labour wards had dedicated newborn care corners with resuscitaires although in some cases these resuscitation tables were makeshift (Figures 5 and 6). Some facilities lacked basic equipment for initial assessment and management of newborns (e.g. pulse oximeters and thermometers), and two primary health centres did not have bag and masks required for resuscitation.

**Table 2.** Availability of equipment for essential newborn care.

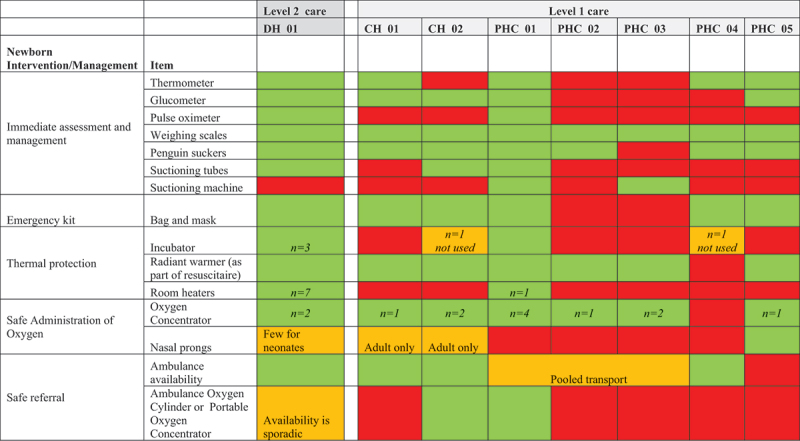
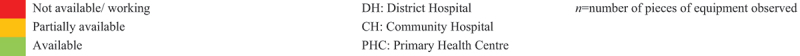

The district and community hospitals had access to a dedicated ambulance for onward referral, and most PHCs reported a shared system of transport with other facilities. Most PHCs did not have access to portable oxygen cylinders to support safe transfer to a higher-level facility.

#### Infrastructure and technology

The district hospital was the only facility with a reliable source of electricity. The other seven facilities reported sustained blackouts of eight hours or more per day. None of the facilities had facility-wide wifi, but most reported wifi for specific projects such as data entry for the District Health Information System (DHIS). All facilities reported the presence of other digital systems and interventions (such as an electronic healthcare record for Anti-Retroviral Therapy), with the exception of one rural centre.

#### Capture and use of data

We found that newborn data were not systematically captured. At facilities providing in-patient care, there were limited data on admissions, diagnoses and outcomes. We found that four out of the eight sites had not captured any newborn data in the last six months.

#### Summary

From these assessments, the adaptation team identified key contextual factors to be considered throughout the adaptation process:

- Site selection for pilot implementation study: a key uncertainty is whether the PHCs can support the intervention given the challenges of irregular power supply, internet connectivity and in some rural centres, a lack of potable water and sterilisation. Any initial pilot of the adapted intervention may be best suited to community hospitals or urban PHCs where these challenges are less pronounced; and at sites closer to the Neotree hub in Lilongwe, so implementation support can be readily provided. Any future pilot should include a pair of linked facilities to enable testing of referral pathways (i.e. a primary urban clinic and one community hospital).
- Adaptation to technology and associated hardware: given the challenges of limited space, power supply and back up, the data dashboard functionality is likely to require adaptation. For example, enabling HCPs to access the dashboards via a tablet rather than displaying data on monitors, which would require a regular source of power and sufficient space.
- Adaptation to the content. The clinical content of the app needs to match the level of care provided at lower-level facilities and requires an embedded algorithm to support HCPs to assess and prepare a neonate for onward referral.

## Adaptation of the Neotree application to fit level 1 newborn care

### Generation of minimum dataset

Following completion of the needs assessments, the adaptation team began the task of adapting the application. The first task was to define the ‘minimum dataset’ of essential demographic and clinical data to be captured for newborns in primary health centres and community hospitals, to meet national surveillance and health system requirements. We developed a prototype digital admission form by incorporating all data fields from the standard paper-based neonatal admission form used in MoH facilities. Additional data fields were incorporated in consultation with the clinical team at KCH, such as information to ensure the safe transfer and continuity of care for a baby referred to a more advanced facility (i.e. vital signs, initial diagnosis and any treatment administered). For the prototype discharge form, we adapted the KCH digital discharge form in order to support the systematic recording of patient outcomes. The final minimum dataset consisted of 280 variables.

### Adaptation of clinical content and decision support

In the absence of national guidelines for management of sick and vulnerable babies in facilities providing level 1 newborn care in Malawi, we relied on paediatricians from the central and district hospitals in Lilongwe to advise on appropriate clinical content. The clinical team first reviewed and adapted stabilisation guidelines from the national Care of the Infant and Newborn (COIN) training course to ensure suitability with PHCs and community hospitals where clinical resources and equipment are limited (e.g. guidance on how to keep a baby warm in the absence of functioning radiant warmers). They then reviewed and revised the diagnostic algorithms currently in use at Kamuzu Central Hospital [Table 3], which were developed using national guidance (COIN) and expert opinion [36].

**Table 3.** Diagnostic algorithms.

1	Low birth weight
2	Prematurity
3	HIV exposure
4	Thermoregulation
5	Hypoglycaemia
6	Jaundice
7	Convulsions
8	Neonatal encephalopathy
9	Respiratory distress
10	Sepsis
11	Congenital abnormalities

Management scripts were adapted to reflect basic case management within level 1 settings, i.e., triage, treat where appropriate and/or prepare for safe referral. The team also developed a referral algorithm to be embedded within the clinical decision support. An example of the adapted guidance for managing a premature baby with respiratory distress is provided in Supplementary File 7.

The clinical team at KCH then tested a series of clinical scenarios to identify gaps in the data capture fields and clinical management pages, and to refine the structure or flow of pages. All revisions to the application were made using the web-based editor. The revised application was now ready for testing with prospective users.

### Usability testing to refine the Neotree application

#### Methods

We conducted two rounds of usability testing using ‘think aloud’ methodology [20,37] with six nurses from a range of lower level facilities consistent with recommendations for iterative user design [38]. Three nurses participated in both rounds, and an additional three nurses participated in the second round (see Supplementary File 5 for participant characteristics). Participants were asked to interact with the application to assess sample clinical scenarios and asked to verbalise their thoughts with regards to:

- how the workflow for the assessment, diagnosis, management and admission/onward referral of a sick baby would fit within their healthcare setting and
- whether the clinical content is a good fit for their setting. And if not, why was this the case, and what should be changed to make it more applicable.

Prompts included ‘what are you thinking as you navigate this page?’, ‘what is causing you to pause’, ‘do you have any suggestions to improve this page?’ Sessions lasted approximately 30 minutes per clinical scenario.

Participants then completed the Systems Usability Scale (SUS) – a 10-item scale which is widely used to generate a composite measure of subjective usability for any given system [39].

#### Results

All feedback from the usability testing was recorded in an Excel spreadsheet (see Supplementary File 6 for excerpts of recorded feedback). The feedback was then discussed among the adaptation team and revisions to the flow, format and content were actioned in the web-based editor following each round of testing. Nurses found the app easy to navigate and the mean SUS score among participants was 79.4 for the first iteration of testing and 92.5 for the second iteration. Both scores indicate above average subjective usability, as a composite score of 68 is the benchmark for ‘average’ usability [40].

#### Data dashboard workshop

We conducted a workshop with five nurses and one clinician (see Supplementary File 5) to explore the format, layout and data elements that might be suitable for a data dashboard in a community hospital or PHC. Given the scant availability of data in these contexts, we had assumed that there would be limited interest in the use and reuse of data. However, participants had clear ideas of what metrics would be useful and how they could use data to drive quality improvement. For example, they requested case fatality rates by day and time of day to explore correlations with staffing levels. While it was beyond the scope of this initial phase of adaptation to co-design a prototype data dashboard, this is planned for future phases. As mentioned earlier, we may have to consider alternative formats to display the dashboard, for example on the hand-held tablets rather than on a monitor.

## Exploring potential barriers and facilitators to implementation of the adapted Neotree platform

### Methods

Following the usability testing, we conducted semi-structured interviews with 10 prospective users to explore the anticipated acceptability of, and potential barriers and facilitators to, implementing the adapted Neotree platform within community hospitals and PHCs. We utilised two behavioural science frameworks in this phase of work. First, the Theoretical Framework of Acceptability which proposes that intervention acceptability, either anticipated or experienced, is influenced by seven dimensions (i.e. self-efficacy, perceived effectiveness, opportunity costs, affective attitude, burden, intervention coherence, ethicality) [41]. Second, we utilised the COM-B framework to explore the potential barriers and facilitators to implementing Neotree. This model proposes that individuals must have the Capability (Psychological and Physical), Opportunity (Physical and Social) and Motivation (Reflective and Automatic) to engage in a specific behaviour or group of behaviours [42]. We developed topic guides ensuring at least one question corresponded to each of the domains of the frameworks (Supplementary file 8).

We invited a purposive sample of 10 HCPs from a range of facilities and seniority to participate (see Supplementary File 5). Potential participants were identified by the adaptation team who had prior contact with the facilities through the needs assessments and usability testing. An independent male Malawian social scientist (KM), with masters training in qualitative research methods, met with invited participants in person, to explain the purpose of the study and answer any questions. All invited participants agreed to take part and provided informed written consent.

KM conducted 10 semi-structured interviews between January and March 2023. Interviews lasted between 40–60 mins. They were conducted in a private room at the clinic or hospital premises in both Chichewa and English. Interviews were audio-recorded, transcribed verbatim and translated into English. The interviewer also recorded handwritten field notes, during and after each interview.

Two social scientists (EW and KM) carried out a combined deductive framework and inductive thematic analysis [43]. Excerpts from the transcripts were first categorised and coded according to each of the domains from the frameworks, and then within each domain we inductively coded and categorised excerpts into themes and sub-themes. Themes related to the TFA were classified as high, low and mixed anticipated acceptability; while themes related to using and implementing Neotree were categorised as facilitators, barriers or mixed. Due to the similarities between the constructs across both frameworks, many themes were classified as representing both COM-B and the TFA. For example, the theme ‘understanding of the purpose and functionality of Neotree’ was categorised as representing both COM-B (Capability-Psychological) and TFA (Intervention Coherence). Analysis was conducted iteratively and collaboratively. Data were managed and analysed using NVivo version 20.

We identified 18 higher level themes. Four were categorised as barriers, six as facilitators and eight as mixed (both barriers and facilitators) [see Table 4 for thematic coding framework with themes, frequencies and exemplar quotes].

**Table 4.** Thematic coding framework.

COM-B Domain	TFA Domain	Theme	Barrier/Facilitator/Mixed	Sub-Theme	No. of participants	Barrier/Facilitator/Mixed	Sample Quote
Psychological Capability	Intervention Coherence	Understanding of the purpose and functionality of Neotree	Facilitator	N/A	10	Facilitator	‘It is a good tool because there is management in the tablet, so l can think of how to manage the baby based on what l learnt in school but with the tablet let’s say if the baby is coughing it will guide us on what to do and at the same time it will also remind us that even after all the assessments and diagnosis what if you choose another management and also it makes you treat the baby as first priority on what the Neotree has suggested’
	N/A	Potential to forget to use Neotree or take shortcuts	Barrier	N/A	3	Barrier	‘l will put it as a priority but we are a busy ward so yes let’s say am very busy l would leave it aside but make sure that l get back to it but l can also easily forget so yes it is bound to happen for Neotree to be forgotten. ’
Physical Capability	N/A	Perceived skills to implement Neotree	Mixed	HCP skills to manage neonates	8	Mixed	‘Most of us after school we don’t go for special training on Neonates, so we have little knowledge on how to manage Neonates and because refreshers are done here and there, we forget most of the steps’ ‘Most of us are conversant with most of the things. And also, the tablet is showing how to do anything so even if its new you can just see and do it l don’t think that’s difficult’
				HCP skills to use digital aids and tools	9	Mixed	‘Yes, they have skills for that because technology has gone far these days, we are using electronics now and again so its not difficult because when they oriented me the next things l was doing alone so it’s that simple.so they can also easily do that and also its asking things directly’ ‘I can’t generalize because as humans we all grasp things differently so some may be slow, but others may grasp it without problems’.
				Perceived skills to interpret and use routine data	4	Facilitator	‘We all have time and we do have the capacity to appreciate data’
PhysicalOpportunity	N/A	Human resources to support implementation of Neotree	Mixed	N/A	7	Mixed	‘Yes, it’s feasible but when it comes to staff shortage then it becomes a challenge, however it’s not always that we have shortages but depending on the roster or excuses from staff then there is a shortage but when we are adequate it can be used properly.’
		Physical resources to support implementation of Neotree	Mixed	Infrastructure (electricity, space, wifi)	9	Mixed	‘… when l look at our setting we need to look at the resources, (…) because it looks like its an electronic thing so we need to have something like continuous backup in terms of electricity because we are prone to lose data also gadgets needs to be charged and we can of course be thinking about backup but those are the things that are missing (…) Internet is another problem because as you know Malawi we are not covered in all areas, so it would be biased because sometimes you would want it to go someplace remote, hard to reach area where it will make sense but our limitation would be there is no internet so these are the things that we need to be thinking about when we want to roll it out.
				Equipment, drugs and supplies	10	Mixed	‘In terms of drugs, we have all that is needed for the Neonates, we have cannulas, pulse oximeters and other necessary equipment’ ‘l think basically it can be the issue of resources (…) we have a lot of nurses here so implementation cannot be very difficult but talking of stationery, pulse oximeters, tablets that would be our challenge’ ‘In our setting we don’t have many resources so Neotree sometimes asks you to do certain things but, you don’t have those resources so it can be tricky’
		Facility security	Mixed	N/A	2	Mixed	‘We have very good security in this area and even the data clerks have their own tablets which they keep here. of course, at every place there can be some people who are there just to disturb but when you talk to them it can all be good’ ‘(…) Theft is theft you know sometimes people take things thinking its something they can use so we should also be able to say if we supply is it going to be safe? especially in the rural areas’
Social Opportunity	N/A	Anticipated institutional support	Barrier	N/A	1	Barrier	‘[this] health centre for me as a centre has a team that is enthusiastic and willing to get new thing onboard, the challenge that they do have which l feel will be mostly is support from management, because when you look at the way they are operating they have got major issues that they have identified but management is not helping so its more of that and so we are looking at whether they will be able to provide internet services think that’s a major thing that am going to say’
Reflective Motivation	Ethicality	Perceived fit with HCP role and values towards digital health care	Mixed	Neotree good fit with HCP professional identity and values	4	Facilitator	‘It will fit, we are moving with technology, and it is dominating (…) we as a HEALTH CENTRE need to move with the world because technology is dominating and we don’t have to cling to old things’ ‘The good thing about Neotree is that its part of midwifery care that we do already so it’s not like it’s a new program no, it’s midwifery and its part of nursing and we are nursers already only that they have removed it and digitalized it.’
	Perceived Interventioneffectiveness	Potential to support and improve management and care of babies	Facilitator	Supports systematic management of babies preventing ‘short cuts’	10	Facilitator	‘It will help the one looking after that baby to manage the baby according to what the baby needs to be treated for because sometimes as a person you tend to forget so this tool as l said it’s a tool it’s going to help us in many ways whereby we can manage properly with the best care because we will be using the Neotree’.
				Supports quality improvement	2	Facilitator	‘It will help us formulate quality improvement plans/projects because data is what speaks for you when you want to lobby for something, for our Neonates, there will be improved Neonatal care and it will minimize mortality and mobility rate.’
		Potential to save HCP time	Facilitator	N/A	3	Facilitator	‘(…)this new development will help our sick Neonates to be helped in time and managed on time without delaying time on seeking solutions from others.‘
		Potential to improve data quality	Mixed	N/A	7	Mixed	‘We need a tablet because it is user friendly and we are missing a lot of data because it gets lost, several files are missing. We need something like Neotree where when you are discharging you enter the data and it is finished.so we are hoping that will be the way forward and the team already welcomed it. “. ”.sometimes digital admissions machines may crash, and we may lose some aagh contents that we entered’
		Potential for negligence	Barrier	N/A	2	Barrier	‘The case of negligence cannot be ruled out, but it is an issue that will require intensive supervision from us. Every system has people who are negligent and lazy, but it will require us supervisors to make sure that such is not happening at our clinic (…)‘
	Burden	Anticipated time burden of using Neotree	Mixed	N/A	10	Mixed	‘My initial impression was that it’s a good application but a bit involving especially with our setting with lack of human resource but with time it’s doable‘ I : From what the providers do already daily here do you think bringing Neotree will complicate their daily duties especially those that do Neonatal care? (…)R: but l do not think so because it’s digital and you just follow, you just click and if it’s a matter of giving drugs that is not feasible but these others are just electronic issue where you go and click and it directs you (…)
	N/A	Confidence among HCP that Neotree can be successfully implemented	Facilitator	N/A	3	Facilitator	‘As you have seen we are a big and busy hospital so with the shortage of nurses it is difficult for a nurse to sit and start collecting data but with Neotree data is collected on the go so am sure the projects will run smoothly’
		Anticipated resistance to implementing Neotree	Barrier	N/A	1	Barrier	‘Gaps may be there, but it can all be an issue of attitude. (…) because it’s a new thing and some people are resistant to change so yes gaps will be there’
	Self-Efficacy	Belief that HCPs have capability to implement Neotree	Facilitator	N/A	8	Facilitator	‘I am very confident because as much as we orient them, and they understand the Neotree they will implement it. We have other systems that were introduced, and they adapted well, and we are still implementing such systems i.e. the ART (antiretroviral treatment) so if it was why not Neotree, so I am confident.‘
	N/A	Intention to prioritise or lead on Neotree implementation	Facilitator	N/A	8	Facilitator	‘l will take it as one of my own and if we have any problems Mondays we have meetings to discuss the previous week so every Monday we need to sit down and see what happened and what we can improve on (…)‘
Automatic Motivation	Affective attitude	Feelings towards potential Neotree pilot	Mixed	Supportive or enthusiastic about potential Neotree pilot	9	Facilitator	‘Yes, the whole idea of the neotree l like it and l can adopt it. what l have noted is that after delivery if you take the sick baby without properly assessing them and you take them to another level without properly assessing them we end up losing them so if we can use neotree am sure we can increase chances of these babies surviving’.
				Reservations towards Neotree pilot	2	Barrier	‘Ok, what l dislike about the Neotree is l have an emergency and then at every single point l must refer to the Neotree, it’s also time consuming ’

### Findings

#### Facilitators

The majority of HCPs understood the purpose and functionality of Neotree [COM-B- Capability- Psychological; TFA-Intervention Coherence] and were mostly supportive and positive towards the idea of piloting Neotree in their facility [COM-B-Motivation-Automatic; TFA-Affective Attitude]. Many suggested that Neotree could support HCPs to deliver improved management of sick and vulnerable babies. In particular, HCPs emphasised how Neotree might support them to give a systematic assessment of a neonate, fill knowledge gaps and ‘prevent shortcuts’ in the delivery of care. HCPs also suggested that Neotree could save time, and ensure better data quality [COM-B- Motivation-Reflective; TFA- Perceived Effectiveness]:

We need a tablet because it is user friendly and we are missing a lot of data because it gets lost, several files are missing. We need something like Neotree where when you are discharging you enter the data and it is finished.

Most HCPs suggested that Neotree was a good fit with their professional identity and values, ‘*we need to move with the world, technology is dominating*’ [COM-B- Motivation-Reflective; TFA-Ethicality]. A few HCPs expressed an intention to prioritise or lead on overseeing Neotree in their facility. Generally, HCPs believed they had the capability to implement Neotree, provided they were given sufficient training and orientation: *‘I am very confident because as much as we orient them, and they understand the Neotree they will implement it’* [COM-B- Motivation- Reflective; TFA- Self-Efficacy].

#### Barriers

Some HCPs expressed concern that there was potential to neglect or misuse Neotree, for example HCPs might skip through parts of the admission scripts or forget to use Neotree during busy periods [COM-B- Capability-Psychological]. One HCP suggested that some nurses and clinical officers may be resistant to change [COM-B- Motivation-Reflective] and one senior manager was concerned about a potential lack of institutional support [COM-B -Opportunity-Social].

For me as a centre [we] have a team that is enthusiastic and willing to get new things onboard, the challenge that they do have which l feel will be mostly is support from management, because when you look at the way they are operating they have got major issues that they have identified but management is not helping …

#### Mixed barriers/enablers

HCPs had mixed views on whether they had sufficient skills to implement Neotree prior to any planned training or orientation [COM-B- Capability-Physical]. While most felt that HCPs would have sufficient digital literacy, some felt that there were obvious gaps in clinical skills:

Most of us after school we don’t go for special training on Neonates, so we have little knowledge on how to manage Neonates and because refreshers are done here and there, we forget most of the steps.

The limited availability of physical and human resources such as staff shortages and limited infrastructure and equipment emerged as a key concern among HCPs from smaller, rural centres but was not so pronounced for those from urban centres [COM-B -Opportunity-Physical]. This included reliable electricity supply, oxygen supplies, wifi and adequate space to treat and manage newborns. A number of HCPs were also concerned that they might not have sufficient drugs and supplies to follow the suggested management plans within the Neotree app:

In our setting we don’t have many resources so Neotree sometimes asks you to do certain things but, you don’t have those resources so it can be tricky.

There were mixed views on whether Neotree might increase the time burden for HCPs [COM-B-Motivation – Reflective; TFA-Burden]. While some anticipated that Neotree would save time and generate efficiencies, others anticipated Neotree might prove time-consuming, particularly at the start of implementation:

My initial impression was that it’s a good application but a bit involving especially with our setting with lack of human resource but with time it’s doable.

In summary, implementation of Neotree was widely anticipated to be acceptable. The majority of potential facilitators to using Neotree within lower level facilities fell within the Motivation domain, while most themes coded as mixed barriers/facilitators fell with the Opportunity domain.

## Stakeholder consultation

In November 2022, we held a series of meetings with key stakeholders including Ministry of Health, United Nations Children’s Fund (UNICEF), Global Health Informatics Institute (GHII), and GIZ (Deutsche Gesellschaft fur Interbnationale Zusammenarbeit) in Malawi. The purpose was to explore a pathway to scale for Neotree outside of tertiary settings in Malawi from proof of concept to adoption, to large-scale roll-out under Ministry of Health ownership and management. We identified a number of partnerships that could be leveraged within a broader health system strengthening approach to achieve this goal. Key opportunities include:

1. Long term Malawi-led project management and oversight via a partnership with GHII;
2. Clinical leadership from Kamuzu Central Hospital and strong support from the Reproductive Health Directorate at the Ministry of Health;
3. Potential to fill equipment and hardware gaps in the Neotree primary healthcare package (e.g. low-cost portable oxygen concentrators) via partnerships with GHII and UNICEF;
4. Potential to ensure aggregate primary healthcare data is integrated with national systems (DHISv2) through continuing partnership with the Digital Health Directorate at the Ministry of Health.

Our ambition to implement an adapted Neotree system at scale is congruent with the aims of the Health Sector Strategic Plan III (2023–2030), namely to ensure equitable access to high-quality services, to improve the competency of the health workforce, and to ensure a harmonised digital health system [44].

## Discussion

We have described the first stages of the adaptation of a digital quality improvement tool (Neotree), which was originally co-designed and developed for HCPs providing level 2 or level 3 newborn care in hospital settings to level 1 care in primary health centres and community hospitals. We have taken a stepwise, participatory and user centred approach to adapt the content of data capture and clinical decision support functionalities of the Neotree application, based on an assessment of the new clinical context, consultations with stakeholders and usability testing with end-users.

Our qualitative research informed by behavioural science frameworks found the adapted version of Neotree was anticipated to be acceptable among HCPs in these contexts. In addition, HCPs were highly motivated to implement Neotree, but key potential implementation barriers included psychological and physical capability (such as skills and knowledge in neonatal care), as well as physical opportunity (such as human resources, equipment and adequate space for newborn care).

Implementation strategies to address these barriers will include providing a basic package of neonatal equipment, essential training in newborn care and resuscitation (e.g. Helping Babies Breathe), training in the use of Neotree, and the recruitment and incentivisation of on-site ambassadors (nurses from the ward) to champion the use of Neotree and troubleshoot technical issues. A key uncertainty is how resource constraints including limited power supply and staffing – common challenges for digital interventions in primary care [45]- will affect future implementation.

The next step in the adaptation process is to conduct a pilot implementation evaluation study, to test implementation feasibility and acceptability in order to refine the adapted intervention and co-develop an implementation model for lower-level facilities. We also hope to generate preliminary data on the effectiveness of Neotree in these settings. We recognise that we are unlikely to achieve similar effect sizes to those observed in tertiary facilities, given the lower volumes of births and newborns requiring intervention [26].

Neotree’s adaptation aligns with Sustainable Development Goal 3 by targeting preventable neonatal mortality in underserved facilities providing level 1 newborn care, complementing global calls for equitable digital health solutions. Future piloting and implementation of our adapted intervention can address critical quality of care gaps by ensuring systematic documentation of every newborn who is assessed in a primary health centre or community hospital (a quality improvement standard in itself) while tailored clinical decision support can aid clinical staff in the initial diagnosis and management of sick and small babies and can guide decision-making for onward referral. However, opportunities to improve clinical outcomes, particularly for those babies who are referred onto more complex care, are likely to be undermined by existing weaknesses within the referral network (e.g. lack of reliable transport, portable oxygen concentrators and dedicated staff for referral). Any future roll out of Neotree at this level is likely to require system-wide investments and solutions to address these weaknesses.

## Conclusion

Using a stepwise user-centred approach, we successfully adapted a digital quality improvement intervention (Neotree) ready for real-world piloting in community hospital and primary health centres in Malawi. During this process, we uncovered acute challenges in the delivery of essential newborn care in lower-level facilities, including a lack of basic clinical training, a lack of essential equipment and scant documentation. This suggests that the provision of newborn care continues to fall short of global standards in Malawi [4]; and signals an urgent need to define and monitor standards of care, including requisite resources and human resource capabilities across all levels of the health system. This standardisation, combined with interventions to operationalise these standards, is critical to ensure that every baby, in every health facility, receives appropriate and timely care, thus maximising their chances for survival and thrival.

## Supplementary Material

Wilson_Supplementary_Files__Final_Sept2025_clean.docx

COREQ_Checklist_EW.pdf

## Data Availability

The data and materials supporting the results will be made available upon reasonable request.
