# Supplementary material for: Adapting a digital quality improvement system (Neotree) for newborn care in primary health centres and community hospitals: a mixed-methods implementation study in Malawi
Source: Glob Health Action. 2025 Oct 23;18(1):2560716. doi: 10.1080/16549716.2025.2560716 (PMC12584896; doi:10.1080/16549716.2025.2560716)
Supplement: Wilson_Supplementary_Files__Final_Sept2025_clean.docx [file ZGHA_A_2560716_SM6410.docx]

**Supplementary Files**

# Supplementary File 1: WHO Levels of Newborn Care

Source: Survive and thrive: transforming care for every small and sick newborn. Geneva: World Health Organization; 2019. Licence: CC BY-NC-SA 3.0 IGO

# Supplementary File 2: Health and Technology Needs Assessment Questionnaire

| Number | Questions | Notes |
| --- | --- | --- |
| 1 | Name of Facility |  |
| 2 | Name of person completing survey |  |
| 3 | Role of person completing survey |  |
| 4 | What is the facility type? (Community Hosp / Rural Hosp, Health Centre-Urban, Health Centre-Semi urban, Health Centre Rural, etc) |  |
|  | *Environmental logistics* |  |
| 5 | What source of power do you have? (ESCOM or Solar)? |  |
| 6 | On average how long does the hospital have no power? |  |
| 7 | Do you have a power backup? |  |
| 8 | How long does the backup last? |  |
| 9 | Is there Wi-FI available? |  |
| 10 | How reliable is the wifi (how many hours per day on average is wifi available?)  What is the bandwidth of the wifi?  What is the coverage (i.e. how much of the facility physically has wifi)? |  |
| 11 | Which network between TNM and Airtel works better within the facility? |  |
| 12 | Is there a printer? |  |
| 13 | Are there any digital systems (computers / tablets) running already? If so, what? |  |
| 14 | Do they have treated water (water board or boreholes)? |  |
| 15 | Do you have any water backup? |  |
|  | *Clinical care* |  |
| *16* | What are the common facilities that refer mothers to this facility? |  |
| *17* | Approximately how many cases per week/month from these facilities? |  |
| 18 | How many mothers deliver on average per month? |  |
| 19 | What are the modes of delivery (SVDs Vs CS)? If CS do they have a theatre? |  |
| 20 | What are the common maternal conditions in pregnancy? |  |
| 21 | How many maternal deaths per month? |  |
| 22 | Partogram/partograph during labour? |  |
| 23 | How many live births are there on average per month? |  |
| 24 | How many stillbirths are there on average each month? |  |
| 25 | How many NNDs on average each month? |  |
| 26 | How many BIDs? |  |
| 27 | Do you, (and then) where do you provide care for sick babies? |  |
| 28 | Neonate patient workflow? (Can you talk me through what happens when you have a sick neonate?) |  |
| 29 | Do you have a baby care unit? |  |
| 30 | How many cots do you have? How often do you go above capacity? |  |
| 31 | How many babies are admitted to the nursery per month?/How many sick babies do you admit per month? |  |
| 32 | Approximately how long does it take you to admit a baby into the facility? |  |
| 33 | What are the common admission diagnosis? |  |
| 34 | The immediate care that they give the child? (Initial care (nurses monitoring, clinician and etc) |  |
| 35 | Where do you get help and advice from if a baby is sick or small? e.g. do you call to ask for help from a specialist? Do you look at the COIN / EDLIZ guidelines (do you have access to COIN / EDLIZ guidelines)? |  |
| 36 | Do you provide kangaroo mother care? |  |
| 37 | Do you provide cup feeding? |  |
| 38 | What equipment does the facility have for the treatment of newborns (e.g. incubators,  thermometers)? |  |
| 39 | What newborn interventions are you able to deliver at the facility? |  |
| 40 | Can you take blood tests from babies at this facility? If so, where do you send them for testing? |  |
| 41 | Documentation: books, register, admission forms, discharge forms, inpatient forms, etc?  (What information do you record about a baby when they are admitted?) |  |
| 42 | Do you have clinical data you can share regarding the main causes of morbidity and mortality in the newborns that you attend to at the facility? |  |
| 43 | Number of referrals? |  |
| 44 | Which facility do you refer to? |  |
| 45 | Referral process (also inquire about the escort/ How would you identify and transfer a sick baby)? |  |
| 46 | Who makes the decision to refer? |  |
| 47 | What paperwork do you use when referring a baby? |  |
| 48 | What happens if a baby dies at the facility (i.e. what are the formal steps that are taken)? |  |
|  | *Staffing/ Administration/Resources* |  |
| 50 | Who is in charge of the facility? |  |
| 51 | What other staff work at the facility? (i.e. doctors / nurses, clinicians) |  |
| 52 | How many staff work at the facility? |  |
| 53 | What are their cadres?/  What is the lowest cadre? What is the most senior cadre? |  |
| 54 | How many HCWs per shift (Labour Ward, Maternity, Nursery)? |  |
| 55 | What training have staff received in newborn care of any kind and/or mentorship programs? |  |
| 56 | What experience have staff had of using printers / tablets / android phones/ Electronic medical records systems |  |
| 57 | Data management – HMIS officer – reporting period – which stakeholders do you share with? |  |
| 58 | QI projects |  |
| 59 | Bed capacity? |  |
| 60 | Basic equipment: Glucometer, thermometer, Pulsometer, heaters, nasal prompts, sanctioning filling? NG tubes? |  |
| 61 | Resuscitator |  |
| 62 | Consumables |  |
| 63 | Equipment available at that hospital? Including ambulance with oxygen concentrators; etc |  |
|  | *General* |  |
| 64 | What are your main priorities in terms of improving the delivery of newborn care at the facility? |  |
| 65 | What are your current IP&C protocols? How is care impacted by waves of COVID-19 infections, if at all? |  |
| 66 | Are there any other challenges you think it is important for us to be aware of? |  |
| 67 | Is there anything else you think it is important for us to know? |  |

# Supplementary File 3: Staffing levels

|  |  | **Level 2 care** | **Level 1 care** | | | | | | |
| --- | --- | --- | --- | --- | --- | --- | --- | --- | --- |
|  |  | **DH_01** | CH_01 | CH_02 | PHC_01 | PHC_02 | PHC_03 | PHC_04 | PHC_05 |
| Staff | Medical Officer | 1 | 1 | 1 | 6 | 0 | 0 | 0 | 0 |
|  | Clinician | 3 | 5 | 5 | 8 | 1 | 1 | 0 | 1 |
|  | Registered nurses | 11 | 4 | 4 | 15 | 2 | 2 | 2 | 0 |
|  | Nurse technicians | 32 | 42 | 36 | 33 | 12 | 10 | 12 | 3 |
|  | Medical Assistants | 0 | 6 | 8 | 4 | 5 | 6 | 6 | 1 |
|  | Community Midwife Assts | 0 | 4 | 2 | 4 | 2 | 2 |  | 1 |
| In-patient capacity | Area for neonatal care | Nursery | Nursery | Neonatal Bay in Paeds ward | Neonatal Bay in postnatal ward | N/A | Postnatal ward | N/A | Postnatal ward |
|  | Number of beds/ cots | 47 cots | 8 beds | 3 beds | 3 cots | 0 | 0 | 0 | 0 |
| Deliveries and admissions | Estimated deliveries per month | 1555 | 481 | 316 | 730 | 115 | 83 | 91 | 50 |
|  | Estimated admissions per month | 400-500 | Unknown | 10 | 5 | N/A | 15 | 5 | N/A |
|  | Admission source | Labour ward, Theater, Postnatal, KMC, Home, Other facilities | Labour ward, Theater, Postnatal, KMC, Home, Other facilities | Labour ward, Theater, Postnatal, KMC, Home, Other facilities | Labour ward, Theater, Postnatal, KMC, Home, Other facilities | N/A | N/A | Labour Ward | N/A |
| Rota for neonatal care | Nurses per day shift | 3 | 1 | 1 | 2 | N/A | N/A | N/A | N/A |
|  | Nurses per night shift | 2 | 1 | 1 | 2 | N/A | N/A | N/A | N/A |
|  | Clinical team per day | 1 consultant | On demand | On demand | On demand | N/A | N/A | On demand | N/A |
| Training in newborn care |  | Some COIN training | Unclear | None | COIN training | Unclear | None | None | None |

DH: District Hospital

CH: Community Hospital

PHC: Primary Health Centre

# Supplementary File 4: Infrastructure

|  |  | **Level 2** | **Level 1** | | | | | | |
| --- | --- | --- | --- | --- | --- | --- | --- | --- | --- |
|  |  | **DH_01** | **CH_01** | **CH_02** | **PHC_01** | **PHC_02** | **PHC_03** | **PHC_04** | **PHC_05** |
| Power supply | ESCOM Power | Yes | Yes | Yes | Yes | Yes | Yes | Yes | Yes |
|  | Power back-up | Genset | Heavy Genset (depends on availability of fuel) / Solar (Non functional) | Heavy Genset (depends on availability of fuel) / Small Genset (for theatre) | Solar (dedicated to the maternity) | Genset (not functional) | Solar (not functional) | Solar (Not functional) | None |
|  | Black-out duration per day | none | A minimum of 8 consecutive hours | A minimum of 8 consecutive hours | A minimum of 8 consecutive hours | A minimum of 8 consecutive hours per day | A minimum of 8 consecutive hours | A minimum of 8 consecutive hours | A minimum of 8 consecutive hours |
| Water | Treated water | Yes | Pumped water | Well with submersive pump (treated ) | Yes | Yes | Yes | Yes | Pumped water (unclear is treated or not) |
|  | Water back up | No | Borehole | No | Boreholes, water reservoirs | 2 water reservoirs | Borehole | Borehole | Borehole |
| Internet Connectivity | Facility-wide Wifi | No | No | No | No | No | No | No | No |
|  | Network | TNM preferred | TNM preferred | TNM preferred | TNM preferred | TNM preferred | TNM preferred | TMN | Both |
| Other systems | Digital Systems | ART EMR, eBRS | ART EMR, LIMS, eBRS, Sepsis | ART EMR | ART EMR, Peri_gen watch  system | ART EMR | ART EMR, ANC, eBRS | ART EMR | None |
| Hardware | Printer Availability | *n*=1 for whole hospital | *n*=1 for whole hospital | Yes | Yes | No | No | Yes | No |

# Supplementary File 5: Profile of Participants

| ID | Designation | Gender | Facility | Usability Testing 1 | Usability Testing 2 | Participatory w'shops | Data Dashboard w'shop | Qualitative Interviews |
| --- | --- | --- | --- | --- | --- | --- | --- | --- |
| 1 | Nurse Midwife Technician | Female | District Hospital |  |  | ✓ |  |  |
| 2 | Nurse Midwife Technician | Female | District Hospital | ✓ | ✓ | ✓ |  |  |
| 3 | Nurse Midwife Technician | Female | District Hospital |  |  | ✓ |  |  |
| 4 | Paediatrician and Neonatal Lead (Registrar) | Female | District Hospital |  |  |  |  | ✓ |
| 5 | Registered Nurse | Female | Community Hospital 01 | ✓ | ✓ | ✓ |  | ✓ |
| 6 | Nurse Midwife Technician | Female | Community Hospital 01 |  |  | ✓ |  | ✓ |
| 7 | Paediatric Clinical Officer | Male | Community Hospital 01 |  |  | ✓ | ✓ |  |
| 8 | Nurse Midwife Technician | Male | Community Hospital 02 | ✓ | ✓ | ✓ | ✓ |  |
| 9 | Nursing Officer | Female | Community Hospital 02 |  |  | ✓ | ✓ | ✓ |
| 10 | Nurse Midwife Technician | Male | Community Hospital 02 |  |  | ✓ |  |  |
| 11 | Nurse Midwife Technician | Female | Primary Health Centre 03 (Urban/SemiUrban) |  |  | ✓ |  | ✓ |
| 12 | Nursing Officer | Female | Primary Health Centre 03 (Urban/SemiUrban) |  | ✓ | ✓ | ✓ |  |
| 13 | Clinical Officer | Female | Primary Health Centre 03 (Urban/SemiUrban) |  |  | ✓ |  |  |
| 14 | Nurse Midwife Technician | Female | Primary Health Centre 04 (Rural) |  |  | ✓ |  |  |
| 15 | Nurse Midwife Technician | Female | Primary Health Centre 04 (Rural) |  |  | ✓ |  | ✓ |
| 16 | Nursing Officer | Female | Primary Health Centre 04 (Rural) |  | ✓ | ✓ |  | ✓ |
| 17 | Senior Nursing Officer | Male | Primary Health Centre 04 (Rural) |  |  |  | ✓ | ✓ |
| 18 | Nurse Midwife Technician | Female | Primary Health Centre 05 (Rural) |  | ✓ | ✓ | ✓ | ✓ |
| 19 | Facility-in-charge | Male | Primary Health Centre 05 (Rural) |  |  |  |  | ✓ |

# Supplementary File 6: Excerpt from recorded feedback from usability workshop

| **Overall comments (18/11/2022)** |  |  | **Legend** |  |
| --- | --- | --- | --- | --- |
| Informal group session, 4 Nurses. App navigation was good, they are clearly well used to smartphones, back buttons, opening apps etc.  Very few issues with functionality or understanding what pages were asking. CDS section required a little more instruction and contextualisation so they could understand why they were being asked to do the tasks | | | Positive feedback |  |
|  |  |  | App issues |  |
|  |  |  | Things to potentially change/address |  |
| **Time point** | **Section** | **HCP feedback** | **Facilitator observations** | **Action** |
|  | Emergency Management: Referral O2 | Portable oxygen sometimes not available | Mentioned that "most" of the time it was available | Caveat management guidance with "Call KCH & follow guidance for transfer" |
|  | Emergency Management: Referral O2 | Escorts not always available - especially at night | Especially in smaller facilities where there is maybe only 1 nurse | Caveat management guidance with "Call KCH & follow guidance for transfer" |
|  | Emergency Management: Hypothermia | Thermometers not always available and Power for rescucitaire |  | Include thermometers in a basic package of equipment (to be supplied to implementing facilities) |
|  | Vital Signs: Head circumference | Don't have measuring tapes |  | Include measuring tapes in a basic package of equipment (to be supplied to implementing facilities) |
|  | Vital Signs: tone | Don't have measuring tapes |  | Include measuring tapes in a basic package of equipment (to be supplied to implementing facilities) |
|  | Patient Information: Birth notification number | Sometimes use Partograph number to track baby from LW |  | Change label to "Birth notification/ Partograph number" |
|  | Symptom Review: feeding | Often NG, tubes, cups & spoons not available |  | Add "unmeasured cup" as dropdown option |
|  | Risk of Covid: mother at risk yes | Not all facilities have nursery |  | Change add "nursery Corner/ Bay" |
|  | Maternal Religion | Add "African Church" |  | add "African Church" |
|  | Maternal Serostatus | HIV treatment and dosage table is no longer current |  | To provide updated table and share |
|  | Assessment of Sepsis | No option to select None |  | Include a None button |
|  | Assessment of Sepsis | Unclear what to do - no instructions on this page |  | Include instruction for the page |
|  | Examination complete | Page says you have completed the Neotree - you haven't |  | Correct the information on the page |
|  | Emergency management: CPAP | CPAP may not be available |  | Update instructions to say "if CPAP available" |
|  | Parent/Guardian info | Shouldn't show if referral |  | Update conditional expression for page to avoid showing if referral present |
|  | Parent/Guardian info | Consent should be verbal |  | Change consent to Verbal |
|  | Parent/Guardian info | Don't have NICUs |  | NICU should be "Nursery Corner/ Bay" |
|  | Parent/Guardian info | Don't have clogs |  | "remove shoes" not clogs |
|  | Parent/Guardian info | Blankets & Jerseys are allowed in PHCs |  | Change the advice |
|  | Parent/Guardian info | Mother and guardians stay with the babies |  | Change the advice |

# Supplementary File 7: Adapted management guidance for Prematurity with Respiratory Distress

| **Management Advice Tertiary Centre** | **Management Advice Primary Health Care centre** |
| --- | --- |
|  | **Baby is pre-term and showed one of the signs/risks of respiratory distress - this baby needs referral** |
| Care must be given to thermoregulation as per preterm guidelines | Prepare this baby for referral according to instructions below. |
| Remember to perform Ballard Score- images are first Neuromuscular followed by Physical maturity assessment |  |
|  | **STABILISATION** |
| **1. Airway and Respiratory support** |  |
| Position the airway in neutral | 1. Assess ABCCCD (Airway, Breathing, Circulation, Coma, Convulsions and Dehydration) and treat emergency signs |
| If saturations < 90% put on oxygen | 2. Ensure baby is on oxygen IF AVAILABLE |
| If distress is significant & > 1kg consider CPAP according to the TRY CPAP algorithm. | 3. Keep warm - place hat and wrap baby |
| Start on Aminophylline if baby is < 34 week or <1.5kg | 4. Review by senior clinician or nurse - if available |
| Consider surfactant if >1000g with severe respiratory distress requiring CPAP and an oxygen requirement >35% | 5. Give stat doses of antibiotics - Xpen and Gentamicin |
|  | 6. If mother is able to express encourage feeding by cup ONLY if baby can tolerate |
|  |  |
|  |  |
|  | **COMMUNICATION& DOCUMENTATION** |
| 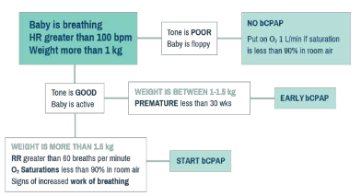 |  |
|  | 1. Inform receiving facility of case summary by phone call or Whatsapp group - discuss availability of portable oxygen & medical escort |
|  | 2. Communicate to guardian intention to refer and reasons |
|  | 3. Attach Neotree print out ready for transit |
|  | 4. Attach all relevant documents/ notes/ checks (glucose, HIV, VDRL etc.) |
|  |  |
| **2. Feeding support** | **MANAGEMENT IN TRANSIT** |
|  |  |
| If breathing 60-80 bpm use cup/OGT | 1. Put baby on portable oxygen IF AVAILABLE |
| If needing CPAP use OGT | 2. Refer in KMC position unless clinical conditions require observation & care in transit |
| If breathing > 80 bpm consider IV fluids | 3. Refer with escort (Nurse or clinician) with BVM equipment - IF AVAILABLE |
|  | 4. Nurse or clinician to bring emergency supplies in ambulance (bag & mask, penguin sucker, dextrose etc.) |
| **3. Get IV access and start antibiotics** | 5. IN PERSON handover by nurse or clinician at receiving facility - if possible |
| Antibiotics are recommended in all preterms with RDS if mother confirmed sepsis or suspected chorioamnionitis OR spontaneous preterm labour OR with 2 or more of the following risk factors for sepsis | Continue to complete the Neotree |
|  |  |
| Risk factors: |  |
| Take blood culture (if possible) before starting antibiotics |  |
| Obtain IV access & commence |  |
| - Penicillin 50,000 IU/kg 12 hourly IV/IM and |  |
| - Gentamicin |  |
| Low birth weight (<2500g) - 3mg/kg 24 hourly IV or IM |  |
| Normal birth weight (>2500g) - 5mg/kg 24 hourly IV or IM |  |
| For senior doctor review for duration of antibiotics |  |

# Supplementary File 8: Interview Guide - Healthcare Professionals

The interview guide was developed to explore all domains of the COM-B model (Michie et al., 2014). The COM-B model can be further elaborated by the Theoretical Domains Framework (Atkins et al., 2017) which maps directly onto COM-B model. We therefore used the TDF to guide the development of the questions.

Warm up questions

What is your position at this clinic? How long have you worked here?

Initial Impressions/Affective Attitude (TFA)/ Intervention Coherence (TFA)

Can you talk me through your initial impressions of Neotree?

What is your understanding of what Neotree is? What it is for? (Prompt: What are the functions / objectives of the Neotree?)

Is there anything that you particularly like or dislike about the proposed intervention?

Skills (TDF)

What are your thoughts on whether HCP at this clinic have the skills to use Neotree?

For example: skills for data entry, taking vital signs, emergency stabilisation and management (prompt content of revised scripts)

What are your thoughts on any additional skills or training that HCPs may need to use Neotree?

Ethicality (TFA); Social/Professional role & identity (TDF)

To what extent do you think it would be safe to use Neotree at this clinic? For staff and for patients (babies)?

To what extent do you feel that the use digital tools fits with HCP roles and responsibilities at this clinic?

To what extent is it fair to ask nurses / HCPs to use Neotree as part of routine care?

Self-efficacy (TFA); Beliefs about capabilities (TDF)

How confident are you that HCP could succeed in implementing Neotree at this clinic?

- Prompt Neotree functionalities (data capture, clinical decision support)

Is there anything you are not so confident about?

Perceived effectiveness (TFA) + optimism (TDF)

In your view, how likely is the Neotree to impact newborn care at this facility?

- How? In what way?

Beliefs about consequences (TDF)

To what extent would there be any benefits/positives of using Neotree?

- Prompt: for you as managers/nurses/ doctors, other colleagues/roles and for patients & families?

Can you envision any drawbacks/negatives? (Prompt: for you as managers/nurses, other colleagues/roles and for patients and families?)

Burden (TFA)

What are your thoughts on whether Neotree is likely to affect staff’s workload?

- In what way?

How much additional effort do you feel it would take to implement Neotree at this clinic?

Goals (TDF)

Compared to other things that HCPs have to do at this clinic, where do you think implementing Neotree would fit as a priority?

Opportunity Costs [TFA]

Do you think there is anything that might be lost by switching to an electronic data capture system?

Environmental context and resources (TDF)

To what extent do you feel that you would have enough resources to implement Neotree?

- Prompt: time, tablets, reliable electricity, WIFI etc..

What additional resources would you need?

Monitoring/ Oversight

What are your thoughts on how best to monitor the implementation of Neotree at this clinic?
